# Understanding teachers' curriculum leadership: a field-mediator mechanism

**DOI:** 10.3389/fpsyg.2026.1663486

**Published:** 2026-04-28

**Authors:** Yi Wan

**Affiliations:** College of Education Ludong University, Yantai, China

**Keywords:** curriculum leadership, curriculum leadership aspirations, curriculum leadership enactment, field-mediator mechanism, influencing factors, teacher leadership

## Abstract

Curriculum leadership is central to curriculum reform in basic education and to the cultivation of students' core competencies. This study adopts a basic qualitative approach to examine how teachers in a centralized Asian context develop curriculum leadership. Data were collected from 21 secondary school teachers in three schools in Yantai, China. The findings indicate a field-mediator mechanism through which field factors and psychological mediators interact to shape teachers' aspirations for curriculum leadership and support the enactment of those aspirations in practice. The study further suggests that the development of teachers' curriculum leadership depends on supportive organizational conditions, particularly small-scale parallel structures and comprehensive evaluation policies.

## Introduction

Teacher leadership has attracted substantial scholarly attention and is widely recognized for its contributions to student development and school reform (Wenner and Campbell, 2017). A broad conception of leadership includes the work of all individuals who contribute to leadership practice, regardless of whether they are formally designated as leaders (Harris and Spillane, 2008). The literature generally distinguishes between two forms of teacher leadership: formal and informal (Liu, 2021). Formal teacher leaders hold administrative or officially assigned roles granted by principals or governments, whereas informal teacher leaders exercise leadership without occupying formal positions (Wenner and Campbell, 2017).

In much of the existing literature, teacher leadership has been associated primarily with formal positions held by selected teachers (Meirink et al., 2019; Muijs et al., 2013). However, curriculum reform and the cultivation of students' core competencies require all teachers to participate actively in curriculum-related matters rather than remain passive recipients of change (Lai and Cheung, 2015; Harris et al., 2020). Teachers have considerable potential for curriculum leadership, as their active engagement with and influence over the curriculum may shape student learning more directly than many other forms of school leadership (Wan, 2024). As the key agents of curriculum leadership, teachers can assume a range of leadership roles throughout curriculum planning and implementation.

### Teacher leadership

Although defining teacher leadership remains contested (Neumerski, 2013), the concept is generally grounded in teachers' capacity to influence others in ways that contribute to school-wide reform, such as fostering a positive school culture, regardless of whether they hold formal or informal leadership roles (Muijs and Harris, 2006). Such influence supports colleagues' professional development (Murphy, 2019) while allowing teachers to retain their primary instructional responsibilities (Harris and Jones, 2020). Because teacher leadership is rooted in the idea of distributed leadership, it creates opportunities for teachers to assume leadership roles at different stages of their professional careers. This process is shaped by dynamic interactions among leaders, followers, and the broader school context across multiple levels (Spillane and Camburn, 2006).

Regardless of whether their roles are formal or informal, teacher leaders exert influence both within and beyond the classroom, and their leadership can take multiple forms. The literature identifies four main leadership functions: (1) leadership in governance, through which teachers participate in school decision-making; (2) leadership of student activities, through which teachers support students' academic development; (3) leadership of operational tasks, such as acting as action researchers to help schools move toward their goals; and (4) leadership of instruction through mentoring and supporting other teachers (Muijs et al., 2013).

Nevertheless, most research on teacher leadership has been conducted in Western contexts (Wenner and Campbell, 2017; York-Barr and Duke, 2004). In many Asian countries, such as China, Japan, and Korea, education is organized through top-down administrative systems. Within these systems, governments at different levels and school principals are typically positioned as leaders, whereas teachers are often expected–and accustomed–to act as followers (Xie et al., 2020). Although some studies suggest that teachers in Asian schools tend to comply with centralized curriculum mandates and rely on traditional pedagogies, others argue that teachers can still exercise curriculum leadership within centralized curriculum systems (Li, 2006). This pattern points to an important limitation in the literature: current understandings are still shaped largely by research conducted in Western and relatively decentralized education systems. Consequently, less is known about how teachers develop as informal curriculum leaders in centralized systems and how such leadership is enabled in practice.

### Curriculum leadership

Curriculum leadership is often defined as the responsibility for developing and improving an appropriate school curriculum. Research on curriculum leadership has been especially prominent in countries with decentralized educational systems (Lo, 2012). From a conventional perspective, curriculum leadership is primarily attributed to school principals, who are seen as responsible for developing and improving the school curriculum (Gumus et al., 2016). More recent perspectives, however, emphasize that teachers and other school staff should also participate in curriculum-related decision-making (Ho, 2010).

As one dimension of teacher leadership, curriculum leadership focuses on curriculum decision-making, vision building, and curriculum development. A curriculum leader is a formal or informal teacher leader who oversees, implements, and enhances the curriculum (Lo, 2012). Curriculum leadership by teachers influences several domains, including students, colleagues, and the whole school. For instance, it can foster student development in various subjects, enhance colleagues' curriculum competencies, and improve the culture and vision of the school curriculum.

Teachers' involvement in curriculum leadership arises from complex interactions among personal and school-related factors. Prior research suggests that principal leadership plays a critical role in promoting teacher leadership and is among the most influential factors shaping teacher motivation (Köse et al., 2024). Principal empowering leadership has also been found to affect teacher agency both directly and indirectly through teacher self-efficacy (Özdemir et al., 2025). A study conducted in Hong Kong further highlighted the interaction between teachers and principals, showing that teacher leadership develops through a collaborative process (Cheng and Szeto, 2016). Organizational structures within schools, such as hierarchical or parallel arrangements, also shape the distribution of leadership and the extent to which different stakeholders participate in leadership processes. Schools characterized by hierarchical structures tend to reinforce principal dominance and constrain distributed leadership (Friedman, 2011). Teacher leadership is further influenced by relationships with administrators and colleagues. In addition to principals, resistant or resentful colleagues may hinder teachers' leadership efforts (Wenner and Campbell, 2017). Personal characteristics also matter. Teachers who are unwilling or unable to challenge traditional hierarchical structures, or who lack confidence, may find it difficult to assume leadership roles (Brosky, 2011). Teacher trust has likewise been shown to have a direct effect on teacher leadership (Kilinc et al., 2021). Moreover, professional development is essential to fostering teacher leadership, and teachers who engage in leadership practices tend to prioritize activities that enhance their professional learning (Sonmez et al., 2025).

Taken together, prior studies indicate that teacher curriculum leadership is shaped by both internal and external factors. Internal factors include teachers' expertise, willingness to lead, and self-confidence (Ho, 2010; Meirink et al., 2019). External factors include the school's organizational structure and culture (Muijs and Harris, 2006), as well as relationships with principals and colleagues (Szeto and Cheng, 2017). Although these studies offer important insights, the specific factors that determine the quality of curriculum leadership remain insufficiently understood. In particular, limited research has examined how different factors interact to shape teachers' curriculum leadership.

Despite these valuable contributions, important limitations remain in the literature. Existing studies have identified a range of personal and organizational factors related to teacher leadership, but they have been less successful in explaining how teachers develop curriculum leadership in centralized education systems, especially in Asian contexts marked by stronger administrative hierarchies. Simultaneously, less is known about how these factors combine to shape teachers' aspiration to assume curriculum leadership roles and how such aspiration is translated into enactment in practice. The present study addresses this gap by moving beyond a descriptive account of influencing factors to examine how personal, relational, institutional, and power-related conditions work together through psychological mediators. This analytical focus is particularly important in the Asian context, where teacher participation in curriculum work is embedded in institutional arrangements and cultural expectations that differ from those commonly described in Western and relatively decentralized systems. Examining teacher curriculum leadership in China therefore not only extends the empirical scope of the literature but also refines current understandings of how curriculum leadership develops under stronger structural regulation.

## Analytical framework

Kurt Lewin's field theory is grounded in two central concepts: field and dynamics. “Field” refers to an individual's life space, whereas “dynamics” highlights the psychological forces that energize and direct behavior. According to Lewin (1951), behavior can be understood only by considering the person and the environment as an integrated whole, captured in the formula B = f_(P, E)_ = f_(LS)_, where LS denotes life space. Life space encompasses the person, the environment, and their interaction. From this perspective, behavior emerges from the configuration of forces operating within the individual's life space.

At the individual level, the internal psychological system provides the energy basis for mental activity and action. Behavior is rooted in internal needs, and when these needs generate psychological tension, a willingness to act may arise. Whether such willingness is translated into action depends on both objective environmental conditions and the individual's subjective interpretation of those conditions. From this perspective, teachers' curriculum leadership aspirations and enactment can be understood as the product of interactions between their internal needs and the environmental factors shaping those needs.

## Methods

This study employed a qualitative multiple-case study design and drew on grounded theory coding procedures, informed by Lewin's field theory, to examine teachers' curriculum leadership in natural school settings. A multiple-case design was appropriate because the study focused on teachers from three schools and sought to develop an in-depth understanding of curriculum leadership across different real-life school contexts. Within this design, grounded theory coding procedures were used as an analytic strategy to identify the factors shaping teachers' curriculum leadership and to explain how these factors influenced teachers' engagement in curriculum-related work. Specifically, this article addresses the following research questions:

1. What factors influence the development of teachers' curriculum leadership?
2. How do these factors shape the formation of teachers' curriculum leadership aspirations and their translation into enactment?

### Participants and data collection

Twenty-one secondary school teachers from three schools in Yantai, China, aged 34–47 years (13 women and eight men), participated in the study. All participants provided informed consent, including permission for their interview data and relevant documentary materials to be used in the analysis and reporting of the study.

Participants were selected through purposive sampling to ensure variation that was relevant to the research questions. The sample size was determined in light of the scope of the study and established recommendations for qualitative research. Three criteria guided participant selection. First, all participants had more than 10 years of teaching experience in China, which ensured that they had accumulated sufficient professional experience to reflect on curriculum-related work. Second, participants occupied one of three school roles that were expected to provide different perspectives on curriculum leadership: (1) subject teachers (ST), (2) subject leaders in non-administrative positions (SLN), and (3) teacher leaders in administrative positions (TLA). Seven teachers were recruited for each role category, resulting in a total sample of 21 participants. This role-based variation enabled comparisons across different levels of involvement in curriculum leadership. Third, participants were drawn from different types of schools in order to capture variation across school contexts and to broaden understanding of how curriculum leadership develops under different institutional conditions. The demographic information collected included gender, age, educational background, and teaching experience. A summary of participants' demographic characteristics is provided in Supplementary Appendix 1.

Semi-structured interviews were used to collect data on participants' career development, professional experiences, and perceptions of curriculum leadership. This method was appropriate because it allowed participants to describe their experiences in depth while also enabling the researchers to explore comparable themes across cases. Each interview was digitally recorded, accompanied by field notes, and transcribed verbatim. The interviews lasted an average of 45 min (range = 40–50 min). To enhance data accuracy and credibility, each participant reviewed the transcript of his or her interview. In addition, documentary materials provided by participants were examined to deepen understanding of their perspectives and school contexts, and to supplement and triangulate the interview data. Sample questions are listed in Supplementary Appendix 2.

### Data analysis

The analysis was informed by Lewin's field theory, which emphasizes that behavior emerges from the interaction between the person and the environment. In line with this perspective, the study examined teachers' curriculum leadership by considering both personal and contextual conditions. Grounded theory coding procedures were used to analyze the interview and documentary data in a systematic and inductive manner, while Lewin's field theory provided the conceptual lens for interpreting the relationships among the resulting categories.

The analysis was conducted in two linked phases. In Phase 1, the aim was to identify the major factors shaping teachers' curriculum leadership. In Phase 2, the aim was to explain how these factors operated through psychological mediators and broader functional fields to influence curriculum leadership enactment.

In Phase 1, the interview and documentary data were analyzed through open coding, axial coding, and selective coding (Williams and Moser, 2019). During open coding, the researchers examined the interview transcripts line by line to identify meaning units and generate initial concepts. Two researchers independently coded an initial set of transcripts and developed a preliminary codebook; differences were discussed until agreement was reached (Scharp and Sanders, 2018; Costa et al., 2020). Axial coding was then used to compare, refine, and connect the initial concepts in order to develop broader categories, while selective coding was used to integrate these categories at a higher level of abstraction (Charmaz, 2014). Guided by Lewin's field theory, these main categories were further organized into three dimensions: the teacher personal dimension, the school dimension, and the society dimension.

In Phase 2, the researchers revisited the coded data to identify the recurrent psychological mediators that participants used to explain why they adopted or resisted curriculum leadership. The researchers then examined how the main categories related to these mediators and established a category–mediator linkage when a category was repeatedly invoked as an immediate reason for action or a barrier to action in participants' accounts. When a category related to more than one mediator, its dominant pathway was identified on the basis of frequency, directness in participants' explanations, and repeated documentation in analytic memos. On this basis, a second-order integration was conducted by clustering categories that shared similar functional roles in shaping the mediators.

To enhance the credibility of the findings, the study triangulated interview transcripts, documentary evidence, and participant transcript review. In addition, two researchers independently coded an initial subset of the interview data and then compared their coding. Any discrepancies were discussed in detail until agreement was reached, and the coding framework was refined accordingly. The researchers also revisited the original data and analytic memos repeatedly throughout the analysis in order to verify interpretations and refine category development. Coding was first conducted on 19 of the 21 datasets to develop the initial explanatory framework. The remaining two datasets were then used for a saturation check and were coded using the same procedures, with constant comparison against the established concept–category–main category–dimension framework. Because these additional datasets generated no new concepts or category relationships and did not require any revision to the framework, the researchers concluded that theoretical saturation had been reached.

### Researcher reflexivity

Given the interpretive nature of qualitative inquiry, the researchers remained attentive to their own positions throughout the study. Both researchers had academic backgrounds in education and were familiar with research on teacher leadership and curriculum reform. Although this background facilitated sensitivity to the meanings embedded in participants' narratives, it also raised the possibility of interpreting the data in ways shaped by prior theoretical knowledge. To enhance reflexivity, the researchers maintained analytic memos, engaged in regular discussion of emerging interpretations, and repeatedly compared developing categories with the raw interview data. In this way, efforts were made to ensure that the analytical framework emerged from the data while remaining critically aware of the researchers' interpretive role.

## Results

### Phase 1 results: categories, main categories, and dimensions

Interview transcripts and documentary materials were read repeatedly to identify meaningful statements related to teachers' curriculum leadership. Open coding generated 45 initial concepts, axial coding condensed these concepts into 22 categories, and selective coding integrated these categories into eight main categories. Table 1 presents examples of the coding process, and Table 2 summarizes the final category structure across the three dimensions.

**Table 1:** Examples of the coding process.

Original materials (sample)	Initial concepts	Categories	Main categories
“I believe English classes should not be limited to the textbook; more practical, English-related lessons should be offered. However, that is the subject leaders' responsibility, not mine.”	Curriculum development seen as others' responsibility	Leadership cognition	Conceptual cognition
“Generating new content in the classroom requires teachers to have a vast knowledge.”	Broad knowledge base	Knowledge accumulation	Professional competencies
“Teachers with excellent professional competencies have stronger self-confidence and self-identity. These traits stimulate their curriculum leadership aspirations.”	Self-confidence derived from professional competence	Self-confidence	Self-efficacy
“She gave me a lot of advice on teaching, and with her help I was able to grow quickly. Our good interaction created a master–apprentice partnership.”	Collegial support and collaborative learning	Fellowship	Interpersonal relationships
“Schools should create an atmosphere for teachers to participate in curriculum leadership and make it the norm.”	Shared atmosphere encouraging participation	Support for teacher development	School culture
“My former principal gave me many opportunities to learn from experts and participate in high-level teaching activities, which was very helpful to my professional growth.”	Principal support for professional growth	Principals' support	Principal leadership
“Schools must develop a democratic and relaxed environment where teachers are allowed to speak and feel heard.”	Democratic environment and voice	Respect and voice	Organizational structure
“Training policies should apply to every teacher, not just a select few, and implementation still requires close supervision.”	Policy support requiring equitable implementation	Policy implementation	Educational policy

**Table 2:** Main categories, categories, and dimensions.

Dimensions	Main categories	Categories	Frequency
Teacher personal dimension	Conceptual cognition (40)	Career cognition	12
		Leadership cognition	18
		Educational cognition	10
	Professional competencies (83)	Knowledge accumulation	33
		Professional skills	35
		Personal qualities	15
	Self-efficacy (18)	Self-confidence	8
		Pressure and drive	10
School dimension	Interpersonal relationships (50)	Fellowship	12
		Teacher–student relationship	18
		Significant others	20
	School culture (53)	Material support	16
		Support for curriculum reform	15
		Support for teacher development	22
	Principal leadership(71)	Principals' support	16
		Principals' guidance	28
		Principals' attitude	27
	Organizational structure (54)	Democratic management	14
		Respect and voice	20
		Intergenerational collaboration	20
Society dimension	Educational policy (20)	Policy orientation	11
		Policy implementation	9

#### Teacher personal dimension

##### Conceptual cognition

Conceptual cognition was an important factor shaping teachers' curriculum leadership. It refers to teachers' understandings of curriculum leadership, their professional roles, and their educational beliefs. Many participants regarded curriculum leadership as the responsibility of administrative leaders or subject leaders rather than ordinary teachers, and some were unable to distinguish curriculum leadership from curriculum management. Such cognitive limitations appeared to weaken teachers' willingness to engage in curriculum leadership.

“*I believe English classes should not be limited to the textbook; more practical lessons should be offered. However, that is the subject leaders' responsibility, not mine.”* (SLN2)

These responses suggest that some teachers still view themselves primarily as curriculum implementers rather than curriculum leaders, which constrains their initiative in curriculum-related work.

##### Professional competencies

Professional competencies also emerged as a key factor. Participants repeatedly emphasized that strong subject expertise and teaching ability provide the foundation for curriculum leadership. When teachers demonstrate professional competence in their core instructional work, they are more likely to gain colleagues' trust and to participate confidently in curriculum-related decision-making.

“*If a teacher wants to play a leading role in curriculum development, the most important thing is to know the lesson content very well and understand what students need to learn.”* (SLN3)

The findings indicate that professional competence underpins both teachers' legitimacy and their confidence in taking on curriculum leadership responsibilities.

##### Self-efficacy

Self-efficacy was another important factor influencing teachers' aspirations for curriculum leadership. It refers to teachers' confidence in their ability to undertake curriculum-related responsibilities. Participants indicated that teachers with stronger self-confidence were more likely to develop curriculum leadership aspirations and to take initiative in practice.

“*Teachers with excellent professional competencies have stronger self-confidence and self-identity. These traits stimulate their curriculum leadership aspirations.”* (ST3)

High self-efficacy appeared to help teachers view curriculum work as an opportunity rather than a risk and to persist when challenges arose.

#### School dimension

##### Interpersonal relationships

Interpersonal relationships constituted an important school-based condition for curriculum leadership. Teachers' curriculum leadership was enacted through interaction with principals, colleagues, and students; therefore, supportive relationships were essential for communication, collaboration, and mutual trust.

“*She gave me a lot of advice on teaching, and with her help I was able to grow quickly. Our good interaction created a master–apprentice partnership.”* (ST4)

The findings suggest that harmonious relationships support both teachers' professional growth and their willingness to engage in curriculum leadership.

##### School culture

School culture also shaped teachers' curriculum leadership. Participants noted that a school atmosphere characterized by shared expectations, supportive activities, and opportunities for participation could encourage teachers to become more involved in curriculum-related work.

“*Schools should create an atmosphere for teachers to participate in curriculum leadership and make it the norm.”* (TLA3)

At the same time, participants stressed that cultural support could be weakened by heavy workloads.

“*I want to participate seriously in these activities, but I am too busy to cope with the workload.”* (ST4)

These findings indicate that school culture promotes curriculum leadership only when teachers are also given sufficient time and space to participate.

##### Principal leadership

Principal leadership was identified as a crucial factor influencing teachers' curriculum leadership. Participants consistently emphasized that principals' recognition, guidance, and support could strengthen teachers' aspirations and create opportunities for curriculum leadership.

“*My former principal gave me many opportunities to learn from experts and participate in high-level teaching activities, which was very helpful to my professional growth.”* (ST2)

“*When our new principal arrived, not only did I develop rapidly professionally, but other teachers in the school also developed considerably.”* (ST3)

The data further suggest that democratic and supportive principals are more likely to foster an environment in which teachers feel able to express ideas, participate in decision-making, and enact curriculum leadership. By contrast, authoritarian leadership may suppress teachers' initiative.

##### Organizational structure

Organizational structure also influenced curriculum leadership. Participants indicated that flatter and more democratic structures encouraged communication, participation, and cooperation, whereas hierarchical structures limited teachers' opportunities to exercise initiative in curriculum matters.

“*To enhance teachers' willingness to lead the curriculum, schools must develop a democratic and relaxed environment where people are allowed to speak, self-confidence is not undermined, respect is given, and teachers feel heard.”* (TLA4)

The findings suggest that rigid hierarchical arrangements may reduce teachers' aspirations for curriculum leadership, while flatter structures create more room for teachers to contribute to curriculum development.

#### Society dimension

##### Educational policy

Educational policy was the main society-level factor shaping teachers' curriculum leadership. Participants noted that policy provides direction for curriculum development and can encourage teachers' involvement in curriculum leadership, especially under the context of curriculum reform.

“*Training policies should apply to every teacher, not just a select few, and implementation still requires close supervision.”* (TLA1)

At the same time, participants pointed out that access to training and professional development opportunities remained uneven.

“*The rivalry is too intense; it's all about a few people and being liked and recognized by the Teaching Fellows. Even if you get pushed up, you might get brushed down in the city.”* (ST4)

These findings suggest that policy support alone is insufficient unless implementation mechanisms ensure fair and broad access to professional development opportunities.

### Phase 2 results: psychological mediators and functional fields

In Phase 2 of the analysis, constant comparison and analytic memoing were used to identify the psychological mediators through which the eight factors shaped teachers' curriculum leadership enactment. Four mediators emerged from the data: awareness, responsibility, fairness, and value. Rather than operating independently, the eight factors clustered into four functional fields according to their dominant roles in shaping these mediators. The Capability Field comprised conceptual cognition, professional competencies, and self-efficacy. The Affective Field was represented by interpersonal relationships. The Institutional Field included school culture, organizational structure, and educational policy. The Power Field was represented by principal leadership.

Awareness refers to teachers' recognition that curriculum leadership is meaningful, necessary, and open to their participation. Participants' accounts showed that teachers were more likely to develop leadership aspirations when curriculum work was made visible and legitimate through both relational and institutional support. By contrast, limited conceptual understanding weakened such awareness. As one participant noted, “curriculum leadership is the subject leaders' responsibility, not mine,” (SLN2) indicating that some teachers did not yet view curriculum leadership as part of their own professional space. Awareness was also fostered through supportive professional relationships. As one teacher explained, “My subject leader supported my professional growth, and that made me realize that curriculum leadership was not something limited to formal leaders, but something teachers like me could also participate in” (ST4). Another participant emphasized that schools should “create an atmosphere for teachers to participate in curriculum leadership and make it the norm” (TLA3). Taken together, these accounts show that awareness is shaped not only by teachers' understandings, but also by relational and institutional conditions that signal whether leadership participation is possible and legitimate. In theoretical terms, awareness was linked mainly to the Affective Field and the Institutional Field.

Responsibility refers to teachers' sense that curriculum leadership is part of their professional role and something they ought to undertake. Participants frequently connected this sense of responsibility with professional competence, self-efficacy, and collegial influence. One teacher explained that “the most important thing is to know the lesson content very well and understand what students need to learn. Only then are we qualified to take the lead in curriculum work,” (SLN3) suggesting that responsibility became stronger when teachers felt professionally capable of contributing. Responsibility was also reinforced through relational support. As one teacher recalled, “She gave me a lot of advice on teaching. Our good interaction created a master-apprentice partnership, which strengthened my confidence in taking on curriculum leadership” (ST4). These findings indicate that responsibility was not simply an individual disposition; it was reinforced when teachers felt competent, confident, and supported in taking curriculum-related initiative. This helps explain why responsibility was shaped mainly by the Capability Field and the Affective Field.

Fairness refers to teachers' perceptions that participation opportunities, procedures, and recognition are distributed in an equitable and transparent way. Participants were particularly sensitive to whether they had voice, access, and equal opportunity in curriculum-related work. One teacher stressed that schools should create “a democratic and relaxed environment where people are allowed to speak and teachers feel heard,” (TLA4) while another pointed out that competition for opportunities was uneven and shaped by being “liked and recognized” by those in authority (ST4). These responses indicate that teachers' willingness to translate aspirations into action depended heavily on whether the institutional and leadership environment was perceived as procedurally just. In this sense, fairness functioned as a key participation condition and was primarily associated with the Institutional Field and the Power Field.

Value refers to teachers' perceptions that engaging in curriculum leadership is worthwhile because it supports professional growth, recognition, meaningful contribution, and identity development. Participants suggested that teachers were more likely to sustain curriculum leadership when they experienced such participation as beneficial and professionally significant. For example, one teacher recalled that a former principal had provided opportunities to learn from experts and participate in high-level teaching activities, which was “very helpful to my professional growth.” (ST2). Another participant noted that stronger self-confidence and self-identity could stimulate curriculum leadership aspirations (ST3). These accounts suggest that value was strengthened when leadership participation brought visible developmental benefits and affirmed teachers' professional worth. Accordingly, value was primarily linked to the Capability Field and the Power Field.

**Figure 1:**
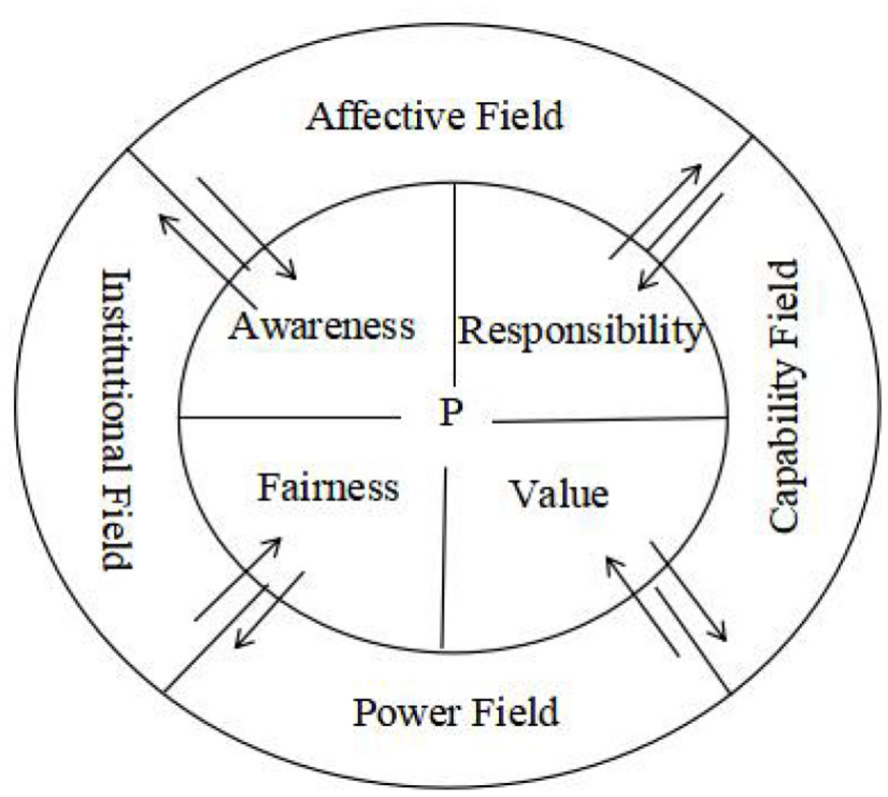
Interaction of the factors influencing teachers' curriculum leadership.

Viewed in this way, the four functional fields did not exert isolated effects; rather, each shaped curriculum leadership enactment through its dominant influence on particular psychological mediators. These dominant field-mediator pathways are illustrated in Figure 1 and provide the empirical basis for the field-mediator mechanism discussed in the next section.

## Discussion

### Framework of influencing factors: from personal to environmental conditions

**Figure 2:**
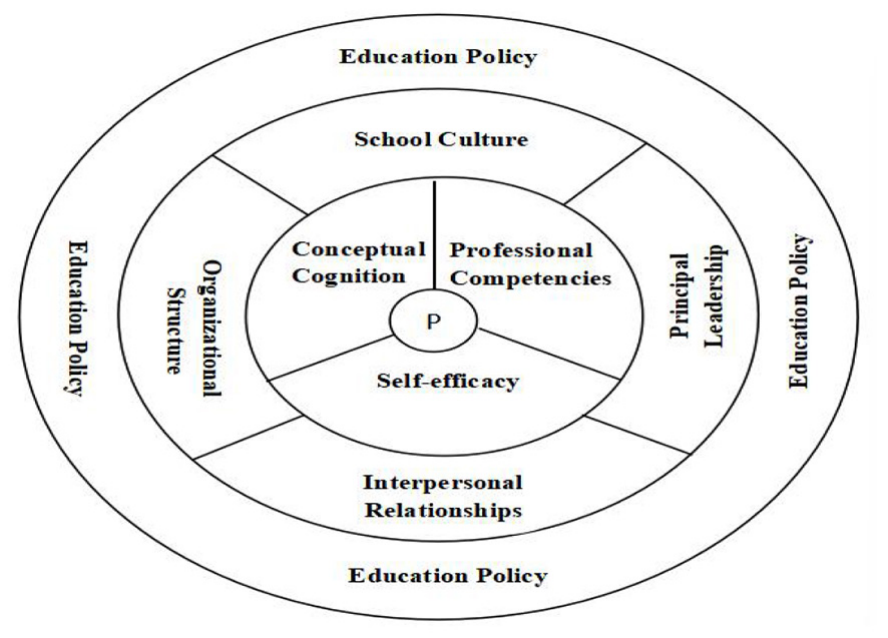
Framework of factors influencing teachers' curriculum leadership.

This study identified eight factors influencing teachers' curriculum leadership: conceptual cognition, professional competencies, self-efficacy, school culture, principal leadership, organizational structure, interpersonal relationships, and educational policy. These findings are broadly consistent with earlier research suggesting that teachers often view curriculum development as the responsibility of formal leaders, which can be a barrier to teachers becoming leaders (Harris and Muijs, 2005), that professional expertise provides an important basis for teacher leadership (York-Barr and Duke, 2004), that principal support is crucial to teacher leadership development (Cheng and Szeto, 2016), that interpersonal relationships help sustain leadership practice (Durias, 2010), and that flatter organizational structures create more favorable conditions for teacher initiative than bureaucratic arrangements (Frost and Harris, 2003). These factors emerged through three stages of grounded-theory coding and were further organized into three dimensions–the teacher personal dimension, the school dimension, and the society dimension–based on Lewin's field theory (see Figure 2).

The relationships among these eight factors can be understood as a nested and dynamic system in which teachers' curriculum leadership is jointly shaped by personal and environmental conditions. At the outermost level, educational policy (*n* = 20) establishes the macro-level boundary conditions that enable and constrain what schools can prioritize and implement. At the middle level, the school dimension serves as the key mediating layer through which policy is translated into teachers' everyday opportunities for curriculum leadership. Specifically, principal leadership (*n* = 71), organizational structure (*n* = 54), school culture (*n* = 53), and interpersonal relationships (*n* = 50) shape the institutional, relational, and cultural conditions under which curriculum leadership becomes possible in practice. Principal leadership and organizational structure provide formal authority arrangements, resource allocation, time structures, and organizational platforms for participation. School culture offers normative support and legitimacy by signaling whether teacher leadership is valued and safe to enact. Interpersonal relationships function as the relational infrastructure through which trust, collaboration, communication, and informal coordination are developed and sustained.

At the innermost level, the teacher personal dimension includes professional competencies (*n* = 83), conceptual cognition (*n* = 40), and self-efficacy (*n* = 18). These factors operate as proximal conditions that directly shape whether teachers are able to lead, willing to lead, and oriented toward appropriate curriculum leadership goals and practices. Together, the three dimensions depicted in Figure 2 illustrate that teachers' curriculum leadership is not the product of any single factor, but rather emerges from the interaction between personal capacities and the broader school and policy environment. This extends previous research by moving beyond the identification of relevant factors to clarify how teacher-related, school-related, and policy-related conditions are structurally connected within the overall field in which curriculum leadership develops.

### Mechanisms of action: from curriculum leadership aspirations to enactment

The findings suggest that teachers' curriculum leadership enactment emerges through the interaction between four functional fields and four psychological mediators. The process can be understood in two analytically distinguishable stages. The first stage involves the generation of curriculum leadership aspirations. Teachers must first recognize curriculum leadership as meaningful and necessary, which reflects awareness, and they must also perceive it as part of their professional role, which reflects responsibility. The second stage involves the translation of aspirations into curriculum leadership enactment. Whether aspirations become sustained action depends on teachers' perceptions of fairness–for example, whether participation opportunities are accessible, procedures are transparent, voices are heard, and the environment provides psychological safety. It also depends on teachers' perceived value of curriculum leadership, including whether participation strengthens professional identity, brings recognition, produces meaningful impact, and supports professional growth. In this process, fairness functions primarily as an entry condition for participation, whereas value functions as a maintenance and reinforcement mechanism that sustains and deepens engagement over time. Accordingly, awareness and responsibility operate mainly as activation mechanisms that generate curriculum leadership aspirations, whereas fairness and value shape whether those aspirations are translated into sustained enactment; successful enactment may in turn reinforce value, responsibility, and awareness, thereby creating a feedback loop.

Viewed in this way, the four functional fields do not exert isolated effects; rather, each shapes curriculum leadership enactment through its dominant influence on particular psychological mediators. The Capability Field operates mainly through responsibility and value. By strengthening teachers' curriculum knowledge, professional competence, and sense of efficacy, it makes leadership feel both feasible and professionally appropriate, thereby reinforcing responsibility. At the same time, accumulated expertise and successful experience enhance teachers' perceptions of leadership as meaningful and worthwhile, thereby increasing its perceived value. The Affective Field operates primarily through awareness and responsibility. Relational trust, collegial support, and emotional affirmation help teachers recognize the meaning and necessity of curriculum leadership, thus heightening awareness, while also fostering commitment by enabling them to internalize shared expectations and obligations. The Institutional Field operates mainly through awareness and fairness. By establishing rules, norms, participation pathways, and organizational expectations, it makes curriculum leadership more visible and legitimate, thereby shaping teachers' awareness of their opportunities and responsibilities. At the same time, institutional arrangements influence whether access, procedures, and participation opportunities are perceived as equitable, and thus directly affect fairness. The Power Field operates mainly through fairness and value. Through authorization, resource allocation, recognition, and voice, it shapes teachers' perceptions of procedural justice and signals whether leadership is supported and worth investing in. Together, these fields explain why the formation of teachers' curriculum leadership aspirations and their translation into enactment depend on whether the surrounding environment enables, legitimizes, and sustains such leadership.

Figure 1 therefore illustrates that when field conditions jointly cultivate awareness, responsibility, fairness, and value, teachers' curriculum leadership aspirations are formed and can be translated into curriculum leadership enactment. Taken together, these findings provide an empirical illustration of Lewin's proposition that behavior is an emergent outcome of interactions between the person and the environment. This extends previous research by moving beyond descriptive accounts of teacher leadership conditions toward an empirically grounded explanatory mechanism.

### Contextualizing the mechanism in china and beyond

China's curriculum reform has gradually shifted curriculum governance from a highly centralized model toward a more distributed system spanning national, local, and school levels. Within this reform trajectory, teachers are no longer positioned solely as implementers of prescribed curricula; they are increasingly expected to participate in curriculum-related decision-making and school-based curriculum development. Teacher curriculum leadership has therefore become an increasingly important capacity for schools seeking to construct, enact, and continuously improve their curricula. In this sense, the reform agenda creates a structural demand for teacher curriculum leadership while simultaneously heightening the importance of the conditions that enable it.

Against this background, the eight factors identified in this study assume distinct context-specific roles through the field–mediator mechanism. The Institutional Field normalizes and structures teacher participation by making curriculum leadership part of everyday school work and by clarifying teachers' roles, eligibility, and participation pathways. In doing so, it strengthens teachers' awareness that they can participate and shapes their perceptions of fairness through access to opportunities, rules, and procedures. The Power Field translates reform demands into principals' concrete practices of authorization and resource allocation. Through these practices, principal leadership directly affects procedural fairness by shaping voice, access, and decision-making transparency, while also enhancing perceived value through recognition, encouragement, and practical support.

The reform process also heightens the salience of the Capability Field and the Affective Field. Teachers' curriculum understanding, professional competence, and self-efficacy provide the immediate capacity base for undertaking reform-related leadership tasks, thereby strengthening responsibility through competence, confidence, and professional meaning-making. Interpersonal relationships, as part of the Affective Field, provide trust, modeling, and collegial support that facilitate entry into collaborative curriculum work, reinforce responsibility through shared commitment, and heighten awareness of participation opportunities. Taken together, these factors explain how reform-driven expectations generate curriculum leadership aspirations through awareness and responsibility, and how those aspirations are translated into sustained enactment through fairness and value in this reform context.

The proposed model should be interpreted first and foremost in relation to the current context of China's basic education curriculum reform, where teachers are increasingly expected to play a more active role in curriculum work, yet participation still unfolds within organizational and cultural norms shaped by seniority, hierarchy, and relational harmony. Under these conditions, teachers without formal or delegated leadership roles may hesitate to identify themselves as curriculum leaders even when they undertake curriculum-related responsibilities in practice. The significance of the model therefore extends beyond China. More broadly, it offers an analytic framework for understanding teacher curriculum leadership in education systems where expanded curriculum responsibility is accompanied by strong institutional regulation, hierarchical authority structures, and culturally embedded expectations surrounding professional roles and relationships. In this sense, the model may offer useful insights for other Asian contexts as well as for non-Western education systems undergoing similar shifts in curriculum governance and teacher participation.

## Conclusion

Lewin's field theory provides the theoretical foundation for understanding the dynamic interaction between individual teachers and their environments. From this perspective, teachers' curriculum leadership enactment emerges from the interplay between personal and environmental conditions, which jointly shape the psychological mediators that activate, enable, and sustain action. Curriculum leadership aspirations and enactment are therefore not merely the product of internal motivation; they are also shaped by how teachers interpret and respond to the field conditions in which they work.

This study identified the main factors influencing teachers' curriculum leadership through grounded-theory analysis and further showed that teachers' adoption or resistance to curriculum leadership can be understood through four recurrent psychological mediators–awareness, responsibility, fairness, and value. On this basis, the eight factors were integrated into four functional fields–Capability, Affective, Institutional, and Power–which together explain how personal, relational, institutional, and authority-related conditions shape curriculum leadership enactment.

Overall, the findings support Lewin's field theory by demonstrating how curriculum leadership enactment is shaped through the interaction between functional fields and psychological mediators. In doing so, the study connects Lewin's theoretical propositions with observable processes in educational settings and shows that teachers' curriculum leadership aspirations and enactment are not simply individual choices, but emergent outcomes of interactions between teachers and their surrounding field conditions.

## Contributions and limitations

### Theoretical and practical contributions

The theoretical contribution of this study lies not in identifying an entirely new set of influencing factors, but in positioning teacher curriculum leadership in a centralized Asian reform context and developing an empirically grounded field-mediator mechanism that explains how commonly discussed influences operate in combination. In doing so, the study extends a literature dominated by Western and relatively decentralized education systems and provides a more explicit explanatory bridge between Lewin's theoretical proposition and empirical data.

The study also offers practical implications for teachers, principals, and education authorities. For teachers, curriculum leadership enactment begins with the development of leadership aspirations. Many teachers remain unfamiliar with the concept of curriculum leadership and continue to regard it as the responsibility of administrators. Such limited conceptual understanding may weaken teachers' willingness to participate. Strengthening teachers' understanding of the meaning, value, and professional relevance of curriculum leadership may therefore help reduce cognitive bias and foster leadership aspirations.

For schools, the findings highlight the importance of organizational and leadership conditions in shaping teachers' curriculum leadership aspirations and enactment. Although flatter organizational structures can help unlock teachers' leadership potential, structural transformation is often gradual, and traditional role expectations may persist even when formal arrangements change. A feasible starting point may therefore be the creation of small-scale parallel structures in which selected teacher leaders guide curriculum-related work and support the participation of their colleagues. Within such arrangements, teachers may have greater opportunities to express leadership aspirations and enact curriculum leadership with less administrative constraint. At the same time, principals can shift from direct control over curriculum affairs toward a more enabling role by offering guidance, professional opportunities, and material support, while also tolerating productive experimentation and trial and error.

For education authorities, the findings suggest that curriculum leadership can also be encouraged and sustained through policy design and evaluation. Policies that explicitly recognize and assess curriculum leadership may help promote its development among teachers. First, evaluation should be differentiated according to teachers' career stages, with different expectations, criteria, and incentives for teachers at different levels of professional development. Second, evaluation should be developmental rather than merely summative. Dynamic assessment tools can support teachers' self-reflection, growth, and professional learning, while comprehensive records of their curriculum leadership development can be used to guide feedback, recognition, and ongoing improvement. In this way, policy can function not only as an external requirement but also as a developmental mechanism for strengthening curriculum leadership enactment.

### Limitations and future research

This study has several limitations. First, curriculum leadership may vary across career stages, age groups, and educational backgrounds. However, the present study was designed to identify shared influencing conditions and common action mechanisms across teachers rather than to make systematic subgroup comparisons. Accordingly, purposive sampling was used and the study focused on experienced secondary school teachers, who were more likely to provide information-rich accounts of curriculum leadership enactment in school-based routines. As a result, the sample is relatively homogeneous in teaching experience and school level, and novice teachers were not included. The transferability of the findings to novice teachers, primary-school contexts, and more diverse career trajectories is therefore limited. Future research could build on the proposed framework by using larger and more diverse samples to examine whether the relative salience and configurations of specific fields and psychological mediators vary across career stages, educational backgrounds, and school contexts.

Second, although this study provides an empirically grounded explanation of teachers' curriculum leadership enactment, its empirical focus is limited mainly to teachers' perspectives and school-based accounts. In practice, curriculum development and improvement are co-shaped by multiple stakeholders. Future research could therefore extend the present framework by incorporating the perspectives of students, communities, and industry partners, thereby generating a more comprehensive understanding of curriculum leadership across broader educational ecosystems.

## Data Availability

The original contributions presented in the study are included in the article/Supplementary material, further inquiries can be directed to the corresponding author.
