# Supplementary material for: Understanding teachers' curriculum leadership: a field-mediator mechanism
Source: Front Psychol. 2026 Apr 28;17:1663486. doi: 10.3389/fpsyg.2026.1663486 (PMC13161107; doi:10.3389/fpsyg.2026.1663486)
Supplement: Supplementary file 1 [file Supplementary_file_1.docx]

**Appendix**

**Appendix1. Participants’ Profile**

| **No.** | **Types** | **Education background** | **Gender** | **Age** | **Experience in teaching** |
| --- | --- | --- | --- | --- | --- |
| 1 | TLA1 | Three-year college | male | 38 | 13 |
| 2 | TLA2 | Postgraduate | female | 37 | 11 |
| 3 | TLA3 | Undergraduate | female | 38 | 12 |
| 4 | TLA4 | Undergraduate | male | 45 | 21 |
| 5 | TLA5 | Undergraduate | male | 43 | 22 |
| 6 | TLA6 | Undergraduate | female | 44 | 20 |
| 7 | TLA7 | Undergraduate | female | 42 | 19 |
| 8 | SLN1 | Postgraduate | male | 38 | 12 |
| 9 | SLN2 | Postgraduate | female | 35 | 11 |
| 10 | SLN3 | Undergraduate | female | 47 | 25 |
| 11 | SLN4 | Three-year college | female | 45 | 21 |
| 12 | SLN5 | Undergraduate | female | 44 | 20 |
| 13 | SLN6 | Undergraduate | male | 43 | 22 |
| 14 | SLN7 | Undergraduate | female | 43 | 22 |
| 15 | ST1 | Three-year college | female | 36 | 12 |
| 16 | ST2 | Undergraduate | female | 34 | 11 |
| 17 | ST3 | Undergraduate | male | 35 | 12 |
| 18 | ST4 | Postgraduate | female | 34 | 10 |
| 19 | ST5 | Undergraduate | female | 40 | 16 |
| 20 | ST6 | Undergraduate | male | 42 | 18 |
| 21 | ST7 | Undergraduate | male | 41 | 17 |

**Appendix 2. Interview Questions**

**Questions for ST and SLN**

1. What is/are your role(s) and scope of work in school curriculum matters? Please describe.

*Probes: What specific activities do you undertake (e.g., curriculum planning, lesson study, assessment design, school-based curriculum development)? How often? With whom?*

1. Were these roles/tasks assigned by the school or initiated by you?

*Probes: How were you selected? What criteria were used? What made you eligible to participate? Did you have a clear entry point or pathway to get involved?*

1. How do you understand teacher curriculum leadership in your school? What practices do you consider as curriculum leadership?

*Probes: When did you first realize you could participate? What signals or arrangements made participation visible and legitimate? To what extent do you see curriculum leadership as part of your professional role, and why?*

1. What do you think about being a teacher leader on curriculum matters?

*Probes: What makes you willing (or hesitant) to take a leading role? Have you ever felt it was “not your place” to lead? What expectations from colleagues/school leaders/policies strengthen or weaken your sense of responsibility?*

1. What are your curriculum leadership aspirations?

*Probes: What specific actions would you like to take (e.g., initiating curriculum improvement, leading lesson study, mentoring peers, coordinating subject/grade curriculum)? Can you describe one episode when you wanted to lead but did not act, and one episode when you successfully acted? What conditions enabled or blocked the aspiration-to-action transition?*

1. What kind of practice does your school or principal adopt to facilitate your curriculum leadership development?

*Probes: How does the principal authorize/empower teachers to lead? What resources are provided? How are opportunities distributed? Do you perceive the process as fair? What makes participation feel worthwhile or valuable? What school-level arrangements support or hinder your participation?*

1. What does the society supply to stimulate your curriculum leadership aspirations or action?

*Probes: What external policies, district requirements, accountability demands, training programs, or society expectations influence your willingness or ability to lead? In what ways do these factors enable or constrain your participation?*

**Questions for TLA**

1. Please evaluate the effects that subject teachers or leaders in non-administrative positions have made in your school.

*Probes: What kinds of curriculum leadership behaviors are most common? What outcomes or changes have you observed? What are the typical strengths and difficulties?*

1. What influences teachers to participate in matters of school curriculum based on individual teachers’ and the principals’ perspectives?

*Probes: Which factors matter most at the teacher level, relational level, school level, principal level, and policy level?*

1. What can be supplied by schools to support and develop teacher curriculum leadership?

*Probes: What structures/platforms, time arrangements, professional learning opportunities, and resource supports are most effective? How are curriculum leadership opportunities allocated and coordinated? How do you ensure equitable access and psychological safety during participation?*

1. In addition to the above, what other conditions stimulate teachers or leaders with non-administrative positions to develop curriculum leadership aspirations or actions?

*Probes: What leadership practices help aspirations translate into sustained action (e.g., authorization/empowerment, workload/time protection, recognition and encouragement, practical support, feedback, and showcasing impact)? How do you handle disagreement, resistance, or conflict to maintain fairness and sustained engagement?*

1. What does the society supply to stimulate non-administrative curriculum leadership aspirations or actions in your school’s teachers?

*Probes: How do policy requirements, district initiatives, and accountability pressures shape teachers’ participation and the school’s support strategies?*
